# Towards an evolutionary theory of the origin of life based on kinetics and thermodynamics

**DOI:** 10.1098/rsob.130156

**Published:** 2013-11

**Authors:** Robert Pascal, Addy Pross, John D. Sutherland

**Affiliations:** 1 Institut des Biomolécules Max Mousseron UMR5247, CNRS–Universités Montpellier 1 and Montpellier 2, CC17006, Place E. Bataillon, 34095 Montpellier, France; 2 Department of Chemistry, Ben-Gurion University of the Negev, Be'er Sheva 84105, Israel; 3 Laboratory of Molecular Biology, MRC, Francis Crick Avenue, Cambridge Biomedical Campus, Cambridge CB2 0QH, UK

**Keywords:** abiogenesis, origin of life, dynamic kinetic stability, systems chemistry, metabolism, irreversibility

## Abstract

A sudden transition in a system from an inanimate state to the living state—defined on the basis of present day living organisms—would constitute a highly unlikely event hardly predictable from physical laws. From this uncontroversial idea, a self-consistent representation of the origin of life process is built up, which is based on the possibility of a series of intermediate stages. This approach requires a particular kind of stability for these stages—dynamic kinetic stability (DKS)—which is not usually observed in regular chemistry, and which is reflected in the persistence of entities capable of self-reproduction. The necessary connection of this kinetic behaviour with far-from-equilibrium thermodynamic conditions is emphasized and this leads to an evolutionary view for the origin of life in which multiplying entities must be associated with the dissipation of free energy. Any kind of entity involved in this process has to pay the energetic cost of irreversibility, but, by doing so, the contingent emergence of new functions is made feasible. The consequences of these views on the studies of processes by which life can emerge are inferred.

## 2. Introduction

The problem of the origin of life can be approached from two directions; from biology back or from chemistry forward. From biology back, Darwin proposed his Doctrine of Common Descent: ‘[P]robably all of the organic beings which have ever lived on this Earth have descended from some one primordial form…’ [1, p. 484]. Woese [2] pointed out that prior to a ‘Darwinian threshold’ being crossed, the earliest life was probably communal with extensive exchange of coded cellular componentry. The origin of this communal life is presumed to have occurred on the early Earth but a precise description of the transition from chemistry to biology will remain out of reach looking back from early living forms [3] because very rudimentary life forms made of unstable organics are unlikely to leave fossil remains. Alternatively, from chemistry forward, the question of the transition may be explored as that of self-organization in chemical systems both through experimental and theoretical approaches [4–6]. This approach connects with the requirement that the process must obey physical and chemical laws in the same way that life has been demonstrated to do [7], which is especially critical when considering metabolism, the way in which ‘*living matter evades the decay to equilibrium*’ [7, p. 69].

The literature of the past 60 years is rich in publications reporting progress through both of these approaches. However, there is still no generally accepted model of the process that could lead to the emergence of life. We share the conviction that general theoretical insights into this evolutionary process can presently be identified without its details having to be disclosed, and we try to summarize the main principles governing this process. We also consider that these views constitute a basis by which systems chemistry [4–6] can expand knowledge in this field unimpeded by historical constraints and potentially able to provide experimental examples of systems manifesting at least some of the features corresponding to those of the living state.

## 3. The improbability of life

**Scheme 1.**
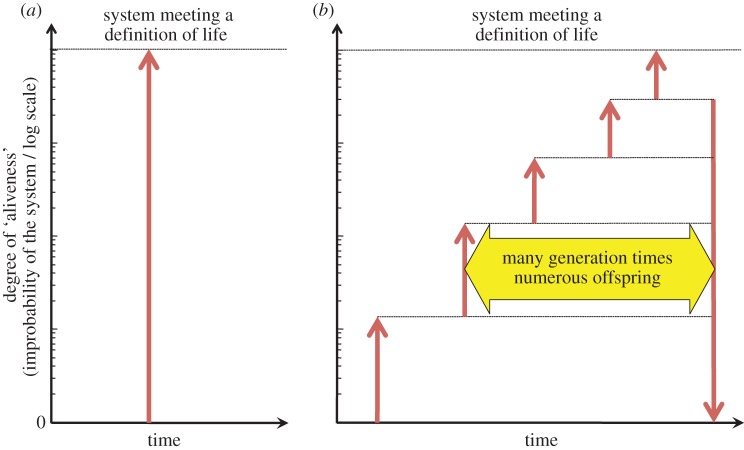
The emergence of life considered as a transition to a highly improbable system. (*a*) Abrupt transition induced by a highly improbable random event in contradiction with the 2nd Law; (*b*) Stepwise process in which intermediate steps (there is in principle no limitation to the number of steps) allow further evolution towards greater degrees of organization on the basis of entities that are capable of reproducing themselves and, therefore, that exhibit a significant persistence before reverting to the unorganized state (right arrow). The choice of a logarithmic scale of improbability for characterizing ‘aliveness’ as the ordinate is purely arbitrary, but in line with the characterization of the emergence of life as an event of low probability.

The elucidation of the double helical structure of DNA 60 years ago [8] provided a molecular explanation for the transmission of genetic information that accompanies cell division. But this breakthrough also prompted a series of discoveries, including that of the genetic code, revealing how nucleic acid sequences are translated into protein sequences using trinucleotide coding of amino acids. At that time, the main bases of biochemistry appeared to be understood and Monod [9] developed a philosophy of biology deduced from all the knowledge that was acquired within two decades that gave a molecular interpretation of the Darwinian theory of evolution proposed a century before. Combined with evolutionary processes, these thoughts provided a profound insight into the most puzzling facets of living organisms, but gave no definitive characterization of the processes by which life originated. As a matter of fact, Monod resorted to a highly improbable random event generating a system possessing essentially all of the basic features of life in one step to explain the origin of life on our planet, and considered it therefore had to be an exception in the universe. If we comply with a probabilistic description of this event, it is possible to set loose limits on its likelihood by considering, for example, the random formation of biopolymeric components, for instance nucleic acids, from their building blocks. The probability of a single sequence of 50 nucleotides among all possibilities corresponds to 1/4^50^ ≈ 0.8×10^–30^ meaning that the exploration of the complete set of sequences over 1 billion years would require the synthesis of more than 4 ×10^13^ different sequences every second. If we consider now that a sequence made up of 100 monomers was needed for a ribozyme to have the wide range of activity allowing the polymerization of the four ribonucleotides, the probability of one single sequence would be reduced to 0.6×10^–60^ and synthesizing all of them in one molecular unit over 1 billion years would lead to the synthesis of a mass of nucleic acid representing several tens that of the Earth per day. These simple virtual calculations clarify how improbable could be the emergence of even a single RNA strand capable of some sort of ribozyme activity within Monod's first living organism, which corresponds to the situation proposed in Scheme 1*a*: a sudden transition from an inert state to the living state. This possibility seems virtually unattainable and it is hardly possible to state as scientific the investigation of a process that is considered as non-reproducible, thus invalidating any experimental study aimed at reproducing the origin of a life form. We thus face a dilemma; either Monod was right, life emerged as a consequence of an event that had almost no chance to occur during the lifetime of the universe, or the emergence of life is not a mere question of the probability of a single event, but a driving force exists—and can thus be discovered—to drive this process through its various stages. So the second possibility—the existence of some driving force governing the evolutionary process—needs to be investigated. It is axiomatic therefore that any *scientific* study of the origin of life must start from the principle that the transition towards life took place through non-zero probability events [10], and according to this principle, life would have emerged stepwise, through states of partial ‘aliveness’, rather than through some single sharp transition, as recently discussed by Bruylants *et al*. [11]. Events, considered individually as having a non-zero, albeit possibly low probability, can then be strung together to constitute an evolutionary process avoiding any violation of this principle. A consequence of viewing the origin of life as a sequence of events rather than a single transition is that ‘a clear-cut frontier between a non-living state of matter and a living system’ becomes impossible [12]. Choosing among the multiple steps in the process and choosing a clear limit separating the living world from inert things becomes a philosophical issue rather than a scientific one (for representative references on the definition of life, see refs. [12–15]).

## 4. Increasing the lifetime of improbable states

However, the possibility that life emerged from a series of intermediate stages requires every one of the stages in this evolutionary process to have sufficient temporal stability so that further improvement can be made (Scheme 1*b*). This condition rules out any possibility that the corresponding states could be populated statistically because the probability of reaching a highly organized state in several steps would correspond to a product of low probability, equivalent to the extremely low probability of reaching the highly organized state in a single step. Indeed, considering that these states are improbable and can only be reached by contingent events, means that their lifetime would be short and could not enable subsequent transitions. *It follows that these stages must be populated with a non-equilibrium distribution and that the disequilibrium state must be constantly maintained.* Any scientific description of the origin of life therefore requires a driving force capable of explaining how these intermediate forms could become stable for long periods of time in a far-from-equilibrium state. The question of the origin of life could then be solved by explaining how states, considered as unstable from a statistical/thermodynamic point of view, independently acquire an alternative form of stability which then allows further improbable changes. One of us introduced a new kind of stability specific to entities that are capable of reproducing themselves called dynamic kinetic stability (DKS) [16–22]. In spite of the possibly short lifetime of the individual components of a collection of similar entities, a reproduction process is actually capable of maintaining their own kind over many generations and of insuring exponential growth to their population under conditions of unlimited resources. Entities capable of being autocatalytically reproduced thus acquire a collective form of stability, quite different from thermodynamic stability, and called DKS to express that fundamental difference [21]. Having these systems present for many generations thanks to a reproduction process, and in many copies thanks to exponential growth, makes a transition to a further degree of organization much less improbable. This means that a physico-chemical driving force for the evolutionary process can be identified, and in that context the role of natural selection becomes clearer—natural selection does not *drive* evolution, but, rather, *directs* it toward systems of increasing stability, the stability associated with persistent replicators, DKS [3,21].

## 5. The kinetic side of dynamic kinetic stability: a specific kind of stability

**Scheme 2.**
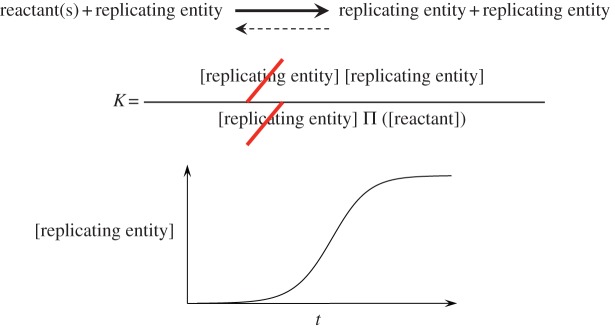
Close to equilibrium, the kinetics of replicator growth levels off and the composition is ruled by the equilibrium constant *K*. As both the forward and reverse reactions are dependent on the concentration of the replicating entity, it can be neglected at equilibrium, meaning that close to equilibrium a replicating system does not behave differently to regular chemical systems. In the exponential growth domain, however, the reverse reaction remains negligible, the irreversibility condition is fulfilled and replication growth becomes unsustainable so that the process is usually limited by the availability of resources.

Studies on replication processes have shown that exponential growth is critical in the selection of the most efficient variants [23–25]. Lifson [26] expressed synthetically the specific power of exponential growth. His analyses demonstrated that two autocatalytic loops, when competing for a single resource provided at a constant rate, evolve towards the extinction of the less efficient one in a similar manner to that for natural selection. This selective behaviour is therefore not specific to living organisms but can be extended to all entities capable of reproduction. It contrasts sharply with the usual first-order chemical processes in which the ratio of product concentrations is constant and determined by the values of rate constants. But it must be emphasized that autocatalysis does not result in growth under close-to-equilibrium conditions [27]. Under conditions close to equilibrium, a replicator (or an autocatalytic process) loses its ability to behave in a specific way (Scheme 2) because the reaction is catalysed in both forward and reverse directions as required by the principle of microscopic reversibility. This means that any autocatalytic cycle or other replication process must proceed *unidirectionally* to display DKS [3]. This line of reasoning thus leads to a conclusion that is identical to that on the issue of the probability of reaching a highly organized state: far-from-equilibrium conditions must be *continually* maintained to observe the specific behaviour of replicating systems. This conclusion is additionally in agreement with one of the main assumptions on self-organization [28, p. 60]: ‘the distance from equilibrium and the nonlinearity may both be source of order capable of driving the system to an ordered configuration’. Although many possibilities of nonlinearity can be responsible for the emergence of dissipative structures in physical systems, chemical transformations behaving nonlinearly are usually limited to systems involving catalytic feedback processes or capable of multiplying themselves, which has been illustrated by studies of model systems (see for instance Wu & Higgs [29]). The nonlinearity induced by the replication/autocatalysis process coupled with the dissipation of free energy associated with the far-from-equilibrium state therefore constituted the main driving force for the emergence of life. The distinction between replicators and autocatalysts is not significant in the context of the present discussion, but has evolutionary consequences that need to be emphasized. Multiplying entities can present different forms and behaviours. A valuable attempt at classifying these systems and precisely identifying the specificity of these forms and their evolutionary potential has been carried out [30]. The analyses presented here do not differentiate between these forms because most of the issues under investigation apply to all forms of multiplying entities.

Exponential growth is conditioned by kinetic equation (5.1) in which first-order terms for autocatalyst [X] and reactant [R] concentrations must be present, though additional terms, in particular ones that express the decay of the catalyst must also be present [25].
5.1
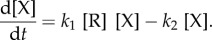


**Scheme 3.**
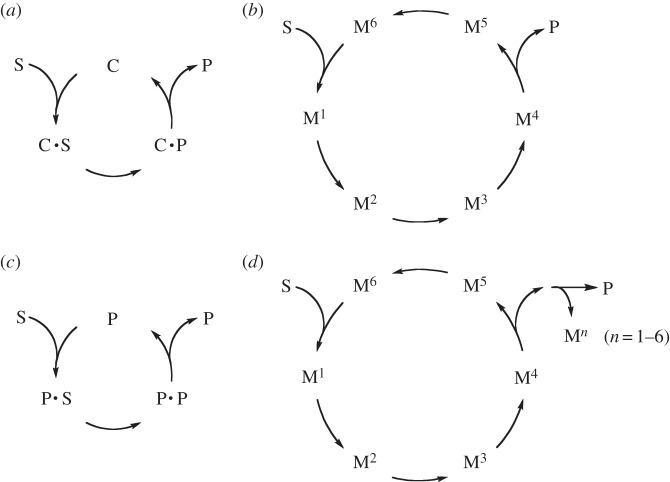
Representation of catalytic cycles (*a*) the usual representation of enzymatic catalysis; (*b*) any reaction cycle with an increased size also gives rise to catalysis with respect to the conversion of substrate S into product P; (*c*) a simple example of autocatalysis; (*d*) autocatalysis can for instance result from the generation of a component (M*^n^*) of a catalytic cycle from a downstream process.

Physically, it means that the process can be represented as a reaction cycle in which the autocatalytic species is recycled and reproduced (Scheme 3). This is the result of the architecture of the reaction network: a simple cycle behaves as a catalyst and a cycle producing a catalyst acting on one of the steps of the cyclic network behaves as an autocatalyst [27]. Autocatalytic sets or networks of reactions have indeed been considered as an essential step in the crystallization of life [31]. Complex networks can be built including cross-catalytic interactions and even hypercycles [23,27,32]. Accordingly, it is important to note that as soon as autocatalysis is present there is no limitation on the complexity of the network, and autocatalysis may result from cooperative or collective behaviour [33]. The network of reactions can involve many kinds of feedback processes including inhibitory ones, such as the formation of an inactive adduct with a product of another loop. There are experimental indications that cross-catalysis can be more effective than direct self-replication of polymer sequences because of product inhibition in simple replication processes driven by pairing [34–36]. Moreover, the cooperation among three mutually catalytic RNA strands seems also to be more efficient than ‘selfish autocatalysis’ [37] giving further insight into the processes by which complexity can develop in evolution [38].

## 6. The cost of irreversibility

**Scheme 4.**
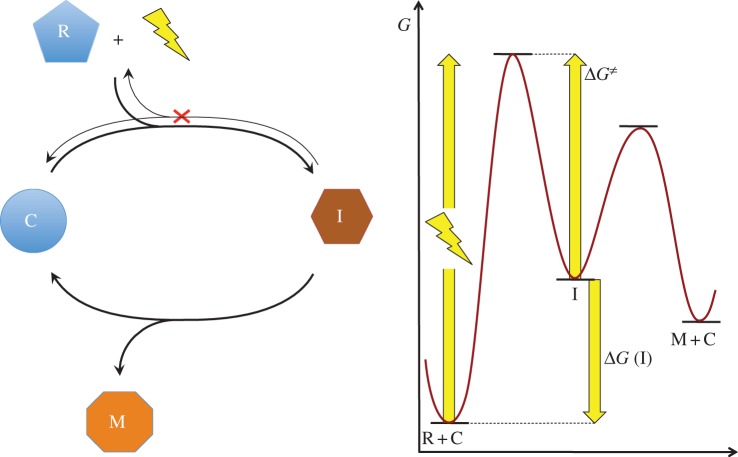
Driving a catalytic cycle (R, reactant; C, catalyst; I, intermediate; M, downstream metabolite) to proceed unidirectionally by coupling with an energy source. Irreversibility requires the waste of an amount of free energy corresponding to the kinetic barrier of the reverse reaction (*ΔG*^≠^). A significant part of the free energy (typically an amount of *ca* 100 kJ mol^–1^ at 300 K for systems with time scales of seconds to years [42,43]) is dissipated so that the loop proceeds unidirectionally, provided that subsequent kinetic barriers remain below that of the activation process. Useful chemical work can be produced from further reactions of intermediate I through its conversion into metabolites (M) but in limited amount (≤*ΔG*(I)) compared with the free energy introduced in the system.

**Scheme 5.**
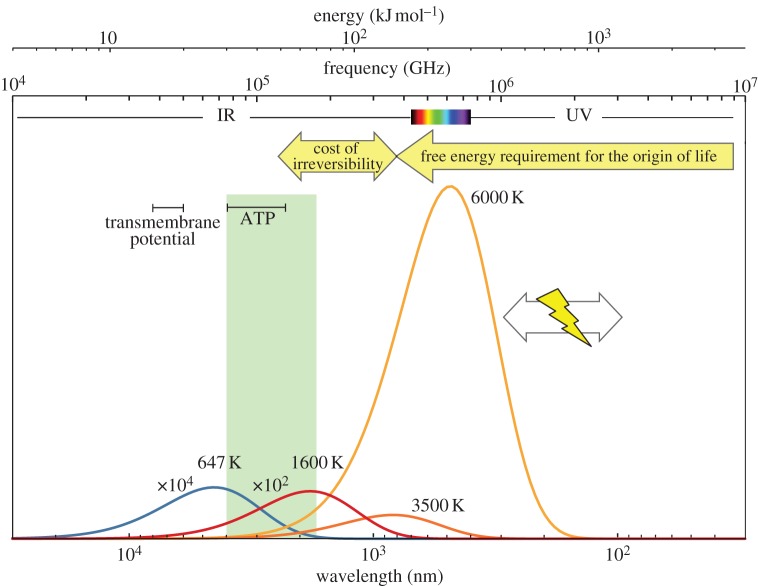
Free energy source requirements in living systems (inspired from the figure introduced by Lineweaver & Chopra [45] with a different perspective). Comparison of different sources of energy available in planetary environments: electromagnetic radiations (correspondence with frequency and wavelength in abscissa), thermal energy (black body radiation curves displaying spectral radiance in ordinate: at 647 K, the critical point of water, blue line; 1600 K, representing typical Hadean magma temperatures red line; and 3500 or 6000 K, dark and light orange lines, surface temperatures of examples of M-stars or G-stars as the Sun, respectively) and lightning (*T ≥*10^4^ K). A much higher potential (*ca* 150 kJ mol^–1^ [42,43]) than the free energy potential of usual biochemicals (green rectangle 30–70 kJ mol^–1^, including ATP) was required to trigger the self-organization of life after taking into account the cost of irreversibility (yellow arrows). Photochemistry induced by UV or visible light (emitted by many stars including a significant part of highly abundant M-stars) complies with the requirement as well as lightning. At higher stages of evolution of life on Earth, the development of metabolic engines allowed the concentration of free energy from less potent transmembrane potentials through chemiosmosis [46]. The development of membrane bioenergetics thanks to rotary ATP synthases and of membranes impermeable to ions made the colonization of new environments possible as well as the use of new energy sources through the exploitation of pH gradients [47]. Overall, these molecular motors operating as energy concentration engines allowed the use of free energy potential of *ca* 15–20 kJ mol^–1^ to drive cell metabolism instead of the almost 10-fold higher potential required to drive early self-organization. By contrast, thermal energy in hydrothermal systems with temperature close to the critical point of water (647 K) fails to comply with the irreversibility requirement for the origin of life.

The formation and the perpetuation of an autocatalytic network, replication loop or any other feature of self-organization require that the spontaneous decay of metabolites involved in the process is slow and that kinetic barriers protect the whole system from rapid evolution towards equilibrium [39–41]. Starting from Eschenmoser's insightful observation and from transition state theory, one of us reached semi-quantitative predictions on the kind of interactions that could support self-organization. Parameters defining the possibility of self-organization were identified as the height of kinetic barriers, the absolute temperature and the turnover timescale of the chemical network [42,43]. At moderate temperatures, allowing for the presence of liquid water, and for timescales expressed in seconds to years, systems based on covalent bonds were inferred as the ones more likely to support self-organization [42–44]. In addition to these conditions, maintaining exponential growth and/or specific selective behaviour of entities capable of self-reproduction requires irreversibility, and a similar kinetic barrier can be introduced for the entire reaction network to proceed unidirectionally [42,43] (Scheme 4). In other words, any self-organizing system has to pay an energetic cost to become irreversible (to prevent the reverse reaction from taking place) through the dissipation of the free energy quantity corresponding to this kinetic barrier. This means that the cost of irreversibility must be expended and cannot be converted into chemical work usable for self-organization. Consequently, a free energy potential equivalent to that of photons of visible or UV light was identified as a semi-quantitative requirement for the self-organization of life at moderate temperatures (Scheme 5) [42,43].

It is noteworthy that the mere assumption that the origin of life was an outcome of a process driven by multiplication of components in reaction networks leads to semi-quantitative conclusions on irreversibility and a relation between temperature, bond strengths and kinetic barriers [42]. It could be represented as occurring in a landscape of free energy in which energy spontaneously flows from high potential sources to low potential products, leading to the recycling of the self-organizing system. However, the idea that the cost of irreversibility has to be paid for is also connected with the idea mentioned earlier, that systems undergoing self-organization at intermediate stages must already be populated in a way quite different from the statistical Boltzmann distribution (see §4).

## 7. The difficulty in quantifying dynamic kinetic stability

How could DKS (the analogue of fitness in the Darwinian theory of evolution) be measured? For a chemical system, the first possibility would be to compare the kinetic behaviour of different systems and the concentration ratios of the products of competing processes, but the limitation of this procedure becomes immediately apparent in that any minute difference in rates between two autocatalytic systems competing for a single resource would result in a qualitative change—the complete extinction of the less efficient autocatalytic system [26]. But other difficulties that undermine any attempt to define a DKS scale arise. Consider for example three different self-reproducing networks A, B and C apparently dependent on a single shared resource. If network A is more DK-stable than B and B more than C, does it necessarily mean that A is more DK-stable than C? This conclusion seems likely if no other *function* is involved. But consider now the possibility that C possesses an activity that interacts negatively with an essential metabolite involved in network A, but not in B, in which case the conclusion could be different. Events occurring through processes independent of the autocatalytic loop may then influence the DKS of the reaction network. A universal scale of DKS seems therefore unattainable from a kinetic point of view simply because evolutionary processes based on the efficiency of replication are opportunistic and the functions that could be recruited to give an advantage are not limited in kind and diversity. Another example of the difficulty in assessing DKS is shown by the recent experiments of the group of Niles Lehman, a network of three cross-catalytic RNA strands seems to be more stable than any single self-replicating one, even when the whole system is allowed to mutate [37].

The measure of a quantity related to fitness in biological systems has constituted the aim of many investigations. Lotka [48] made probably one of the first attempts in this direction during the very early development of biophysics by proposing ‘the principle of maximum energy flux’ stating that natural selection will operate to increase this flux. However, he rapidly understood the limits of this approach [49]. This issue is therefore related to the identification of extremum principles that could rule evolution. Classical thermodynamics can predict the direction of evolution of a system towards the equilibrium state where entropy reaches a maximum value because all the microstates accessible to the system are populated according to Boltzmann's distribution law. However, thermodynamics never predicts the time evolution of a system. In the same way, the application of extremum principles to far-from-equilibrium processes is subject to discussion or requires specific condition [50,51] although it is worth noting that an attempt has been made to use this approach in origin of life studies [52]. Furthermore, attempts to understand life and its origins through a thermodynamic description, including far-from-equilibrium approaches (see Morowitz [10] for an insightful attempt of this kind), are likely to solve only part of the problem because the stability principle supporting life is of a kinetic nature and relies on the persistence of multiplying entities rather than on regular thermodynamics. Proposing the hypothesis of an unknown thermodynamic principle runs into the same difficulty [53].

With respect to the origin of life, it would be logical, considering a single replicating system that grows at the expense of a limited resource, that the variants that are selected for are those which tend to increase the overall replication rate, increase the population of replicating entities and deplete more efficiently the resource so that less efficient variants would be driven to extinction. This observation suggests that the effect of increasing DKS would mainly result in an increase in the chemical flux, but that would only be true for an isolated set of variants of a replicating system. Any improvement in DKS corresponds to an increase in the energy flux diverted from spontaneous linear processes by the presence of replicators. Thus any increase, either in the rates of the rate-determining process or in their efficiency, will increase the population of replicators thereby increasing the flux. But it must be taken into account that from a chemical point of view, every intermediate in the replication loop can be considered as an energy resource for other systems. Therefore, introducing a predator system would lead to a *stable* configuration (i.e. without the possibility of spontaneous reversion to the former state). A reduced reactant flux could then be observed without modification of the replication loop (the notion of DKS may in this case include periodic variations predicted for predator–prey systems by the Lotka–Volterra equations). This example shows that external parameters are likely to influence the flux of reactants, and thus the behaviour of a replicating system, so that no parameter characterizing its DKS can be found independently of the environment in which the system is embedded, which poses a never-ending issue about the boundaries of the system to be considered. Additionally, the question of characterizing ecological systems through a version of DKS that would be capable of integrating multiple interactions is far beyond the scope of this work.

## 8. The utility of the dynamic kinetic stability concept

Many researchers might question the value of DKS given the difficulty in making it fully quantitative. However, the concept does express the kinetic driving force acting on the evolution of entities able to reproduce themselves. It also expresses the opportunistic nature of selection between these kinds of systems—reproducing themselves in an autocatalytic or replicative way—which proceeds in a given environment, and which includes physical sources of energy and any form of nutrient. Many factors can therefore influence the evolution of a reproducing entity, including changes in previously unrelated factors, as well as variations owing to sequence modifications during the replication of biopolymers. The DKS concept only expresses the fact that when these kinds of systems compete, one of them will tend to drive the others to extinction because of differences in reaction kinetics. It does not, however, allow any prediction of the result of this selection process. The result is context-dependent. In fact, evolution towards subsequent states cannot generally be predicted by any extrapolation of the present behaviour because the evolutionary success is highly dependent on the occurrence of previously unrelated contingent events. But this contingent possibility of intervention of additional functions that disallows the elaboration of a predictive DKS scale is precisely the source of an evolution process towards higher complexity which characterize living systems [38]. This is consistent with the view that organizational closure, function and complexity have a close relationship in biological systems [54]. On the other hand, indications from previous reports [16–22] and supported by our present analyses (Scheme 1) have shown that a form of stability that is different from thermodynamic stability is needed to understand how far-from-equilibrium chemical states may have gained a form of persistence, thereby opening the possibility of self-organization toward life. Identifying DKS as a fundamental stability kind in nature is then a necessary step in understanding the emergence of life.

## 9. Conclusion

Irreversibility and the kinetic power of reproduction seem to be, at least in principle, sufficient to allow the emergence of life and there is no need to seek out some hitherto unknown physical law to explain the origin of the specific behaviour associated with living organisms. The connection of energy gathering systems and replicator dynamics must be considered as essential for the origins of life [55]. We have demonstrated here how these two features are so intimately related that they cannot be considered independently. They can be considered simply as the thermodynamic and kinetic aspects of the behaviour of replication/autocatalysis. The hypothesis that the origin of life may have proceeded stepwise through states of partial ‘aliveness’, which is the obvious consequence of a scientific view that a sharp transition is not physically realizable because of its improbability, is therefore sufficient to outline the nature of the process leading to life as we know it, one grounded solely on established laws of physics and chemistry. Many studies since Eigen [27] and Gánti [56] have demonstrated the importance of autocatalysis and replication; other studies have referred to far-from-equilibrium thermodynamics for explaining self-organization [28]; yet others have proposed that some kind of selection was needed in the chemical world before the emergence of evolution so that ‘*it is meaningless to draw a strict line between the two worlds*’ [57]. Our goal was to unify these approaches and connect them in a logical way so that a synthetic view of the origin of life process can be proposed; a view which is readily understandable on the basis of physics and chemistry.

Starting from the axiomatic principle that a transition to life is not physically and statistically impossible, and choosing a temperature compatible with the presence of liquid water, we end in a semi-quantitative representation consistent with life as we know it, which is based on covalent bonds and largely dependent, directly or indirectly, on visible light from the Sun (Scheme 5). This representation of the origin of life process has then the capability of explaining the living world in a consistent way. There has been a lively discussion on the opposition of Monod's views considered above and de Duve's ‘cosmic imperative’ [58]. Contrary to deterministic views, the ideas developed here do not allow any assessment of the level of probability of life and its emergence, nor any prediction of its evolutionary path. Rather they support the idea that spontaneous self-organization of systems manifesting many of the features of living beings is a *reasonable possibility in the physical world*, provided that several conditions are met. Indeed one could reasonably expect that these ideas will likely become heuristically important for experimental studies in systems chemistry. Such studies may include research into closure of reaction networks, enabling them to become catalytic or autocatalytic, or to express new functions. The definition of requirements for the origin of life, such as the need to pay the cost of irreversibility, is also useful in selecting processes potentially of interest among a wide range of possibilities. For instance, many of the studies carried out in order to identify catalytic cycles have begun with the analysis of present-day biochemical cycles of carbon fixation (for a critical overview, see refs. [59–61]; for examples of specific pathways, see Morowitz *et al*. [62], Wächtershäuser [63], Huber *et al*. [64], Martin & Russell [65]) and success in these investigations is likely to be severely limited by the fact that many of these processes do not comply with the condition of irreversibility and can therefore be ruled out as processes driving self-organization. For instance, carbon fixation from CO_2_ using the reducing power of hydrogen or that of less efficient reducing agents is not sufficient to bring about irreversibility [44]. Alternatively, this limitation would no longer be present when starting from an energetically richer inorganic carbon precursor for example HCN, instead of CO_2_, possibly explaining its relationship with the constituents of the reductive citric acid cycle [66] and the efficiency of the formation of a variety of precursors from HCN [67,68], which together suggests considerable potential for this process. Another direction of potential interest could be to seek the emergence of autocatalytic cycles in combinatorial mixtures of prebiotically plausible reactants and activated reagents or energy sources. Selecting processes in which free energy dissipation complies with the threshold for paying the cost of irreversibility could be helpful to these investigations by limiting the number of possibilities. Provided that transient species can be involved in further processes before being destroyed, photochemical steps seems particularly promising in this regard because irreversibility can be introduced directly in the environment of interest without the need to resort to the migration of activated species from a location in which they are formed to that of the self-organizing system. On the contrary, a translocation of metastable species (energy carriers) is mandatory for processes initiated from many other energy sources acting non-selectively on simple inorganic precursors as well as being harmful to species resulting from the self-organization process (e.g. heating to high temperatures or lightning). Lastly, the analyses developed here clearly show that investigations in the field of the origins of life should be divided in two categories:

— The first category is related to the formation of organic matter from inorganic sources of carbon and energy. The fact that these processes could be exergonic or close to equilibrium is of no concern provided that organic building blocks are formed. Processes of this kind have been identified in interstellar space, in the atmosphere of planetary bodies and in hydrothermal systems found at the bottom of the oceans. But it is known since Wöhler's synthesis of urea that organic matter is not specific to the living world and that building block synthesis is therefore not sufficient, though presumably necessary, for the emergence of life.
— The second category is more demanding because the corresponding processes must involve the dissipation of energy, and therefore the cost of irreversibility has to be covered in order for self-organization to take place. Regardless of whether the process starts from abiotically available building blocks and energy sources present in the environment, or directly from simple activated chemical species (capable of producing chemical work and produced through a pathway complying with the threshold for irreversibility), the essential condition for self-organization is that replication or autocatalysis can exhibit their special kinetic features allowing the system to become dynamically stable, so that transient improbable states become persistent over longer periods opening the possibility of subsequent change.

As a final comment, it is most satisfying to note that processes governing transformations in both inanimate and animate systems can be couched in stability terms, each underpinned by its unique mathematical logic. There is thermodynamic stability, the stability kind that dominates the regular chemical world, whose essence has been understood since Boltzmann, and which involves the drive of physico-chemical systems toward more probable states. And contrasting with this familiar stability kind is DKS, a stability kind specific to persistent replicating systems and derived from the dynamic persistence associated with exponentially driven self-replication. Ultimately, the essence of biology should become explicable by the way in which these two quite distinct stability kinds, each resting on its particular mathematical logic, are found to interrelate.
